# Intraventricular SEEG and laser ablation for the treatment of infantile spasm: Technical note

**DOI:** 10.1002/brb3.3184

**Published:** 2023-07-26

**Authors:** Xinghua Xu, Qun Wang, Yining Zhao, Xin Xu, Zhichao Gan, Shiyu Zhang, Xiaolei Chen

**Affiliations:** ^1^ Department of Neurosurgery The First Medical Center of Chinese PLA General Hospital Beijing China; ^2^ Department of Neurosurgery Erlangen‐Nuremberg University Hospital Erlangen Germany

**Keywords:** epilepsy, infantile spasm, laser ablation, ventriculoscope

## Abstract

**Objectives:**

Infantile spasm (IS) is an epileptic encephalopathy with ongoing neurological damage due to seizures and epileptiform abnormalities. Epilepsy surgery is considered for children refractory to drug therapy, especially when there is a focal brain lesion. In this study, we investigated the feasibility and efficacy of intraventricular stereotactic electroencephalography (SEEG) and laser ablation for the treatment of IS children with focal brain lesions.

**Methods:**

We performed the first reported study using ventriculoscopic laser ablation to treat IS. Seven IS children with drug‐resistant epilepsy and definite encephalomalacia on brain magnetic resonance imaging scan were included in this study. Ablation was performed after confirmation of epileptiform discharges by SEEG under the surveillance of ventriculoscope.

**Results:**

The median follow‐up time for the cohort was 3.1 years and 86% (6/7) of the children had an Engel class ≤III epilepsy at the final follow‐up. Five (71%) children had a reduction in seizure medication usage, and the other two were on the same amount as preablation. None of the children experienced serious new neurological deficits. Laser ablation might result in seizure freedom by destroying the local brain network and blocking the spread of abnormal discharges.

**Conclusions:**

Intraventricular SEEG and laser ablation was feasible and effective for the treatment of IS. Further studies are warranted.

## Graphical abstract

infantile spasm; laser ablation; ventriculoscope; epilepsy

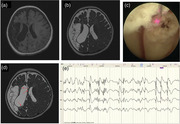

## 1 INTRODUCTION

Infantile spasm (IS), also known as West syndrome, as a specific type of infantile seizure, was first reported by William James West on his own son James Edwin in 1841 (West, 1841). As a severe, age‐related generalized epilepsy syndrome, IS is characterized with an early age of onset, a special form of convulsion, a highly disorganized multifocal electroencephalography (EEG) pattern called hypsarrhythmia, and developmental delays (Pavone et al., 2020). IS is an epileptic encephalopathy with ongoing neurological damage due to seizures and epileptiform abnormalities (Wilmshurst et al., 2017). Though long recognized, IS is still a cause of significant morbidity in children. Early diagnosis and timely treatment of IS is important, nevertheless, that is often delayed due to the difficulty to recognize and diagnose. The identification of a structural cause has particular importance as it may indicate possible curative surgery.

The etiology of IS remains diverse and hundreds of causes have been reported, including focal and diffuse pathologies (Osborne et al., 2010). Perinatal insults, perinatal asphyxia, malformations of brain and cortical development, chromosomal disorders, inborn errors of metabolism, and single gene disorders are the common etiologies of IS (Chopra, 2020; Specchio et al., 2020). It is difficult to explain each single event causing IS and about one fifth to one third of patients do not have an identified etiology through all the investigations (Pavone et al., 2014). The disruption of normal brain neuronal/interneuronal networks may explain the characteristic EEG pattern of hypsarrhythmia. Hypsarrhythmia is likely generated in subcortical structures as the parietal and occipital cortex, and the focal lesions may spread down to the basal ganglia.

There is still a long way to go for the treatment of IS. Currently, adrenocorticotropic hormone (ACTH), corticosteroids, and vigabatrin are considered the standard treatments for IS (Kelley & Knupp, 2018). However, how ACTH works is entirely unclear, and the dose, formulation, and duration of treatment are still controversial. Vigabatrin is an antiepileptic agent that increases gamma‐aminobutyric acid (GABA) levels in the brain by inhibiting GABA transaminase. Outcome measures vary across studies; thus the comparison of hormonal treatment and vigabatrin is difficult (Lux et al., 2004). Combination therapy of hormone and vigabatrin was reported to have a higher response rate than the hormone alone therapy (O'Callaghan et al., 2017). Ketogenic diet, antiepileptic drugs such as topiramate in high doses (10 mg/kg/d), cannabidiol, pyridoxine (vitamin B6) in high doses, and transcranial direct current stimulation were also tried for refractory IS. Epilepsy surgery is a choice for children refractory to drug therapy, especially when focal brain lesions are revealed. Given the potential morbidity associated with epilepsy surgeries, significant efforts have been devoted to improving the safety profile of these surgeries. Laser ablation is a minimally invasive technique that offers an alternative approach to open surgery for eliminating epileptogenic zones through inducing thermocoagulative necrosis (Barba et al., 2017). The successful use of laser interstitial thermal therapy (LITT) to treat epilepsy has been described, harnessing its invasive nature to improve the safety of epilepsy surgery (Lewis et al., 2015).

Different from LITT, the probe of which was placed with the assistance of frameless or frame‐based stereotactic techniques (Bown, 1983), ventriculoscopic laser reached the target through the instrument channel of the ventriculoscope rather than through stereotactic puncture. In addition, the ablation was done under the observation of ventriculoscope rather than under the monitoring of intraoperative magnetic resonance imaging (MRI). In this study, ventriculoscopic laser ablation was attempted to treat IS children with definite focal lesions on brain MRI. The targeted ablation location was confirmed with abnormal epileptiform discharges recorded on intraventricular stereotactic EEG (SEEG). To our knowledge, this is the first study to investigate the feasibility and efficacy of intraventricular SEEG and laser ablation for the treatment of IS with focal brain lesions.

## 2 METHODS

### 2.1 Equipment and methods

In this retrospective study, we used the Ligenesis‐MY100C (Radium Health Science and Technology) Nd:YAG laser to ablate suspected epileptic foci under the surveillance of LOTTA 6° ventriculoscope (Karl Storz) in drug‐resistant IS children with definite encephalomalacia on brain MRI. The LOTTA ventriculoscope has a sheath diameter of 6.8 mm, with one working channel (diameter 2.9 mm) and two side channels (diameter 1.6 mm). The Ligenesis‐MY100C could generate contact laser with a wavelength of 1064 nm and had specially designed flexible optic fiber for endoscopic neurosurgery. As the smallest diameter energy device, the fiber diameter is only 0.6 mm, which can easily go through the ventriculoscope working channel (diameter 2.9 mm). The contact laser targets on hemoglobin with an action depth only 0.5 mm. Laser output is pulsed, with a thermal damage radius less than 1 mm, bringing the effective protection of the surrounding brain tissue. Contact laser may reduce intraoperative bleeding by completing the process of cutting, gasification, coagulation, and hemostasis simultaneously. With no glare and a thin carbonized layer, contact laser operation has a clear surgical field. Bleeding of arteries with diameters less than 2 mm and veins with diameters less than 3 mm can be accurately stopped with the contact laser.

**FIGURE 1:**
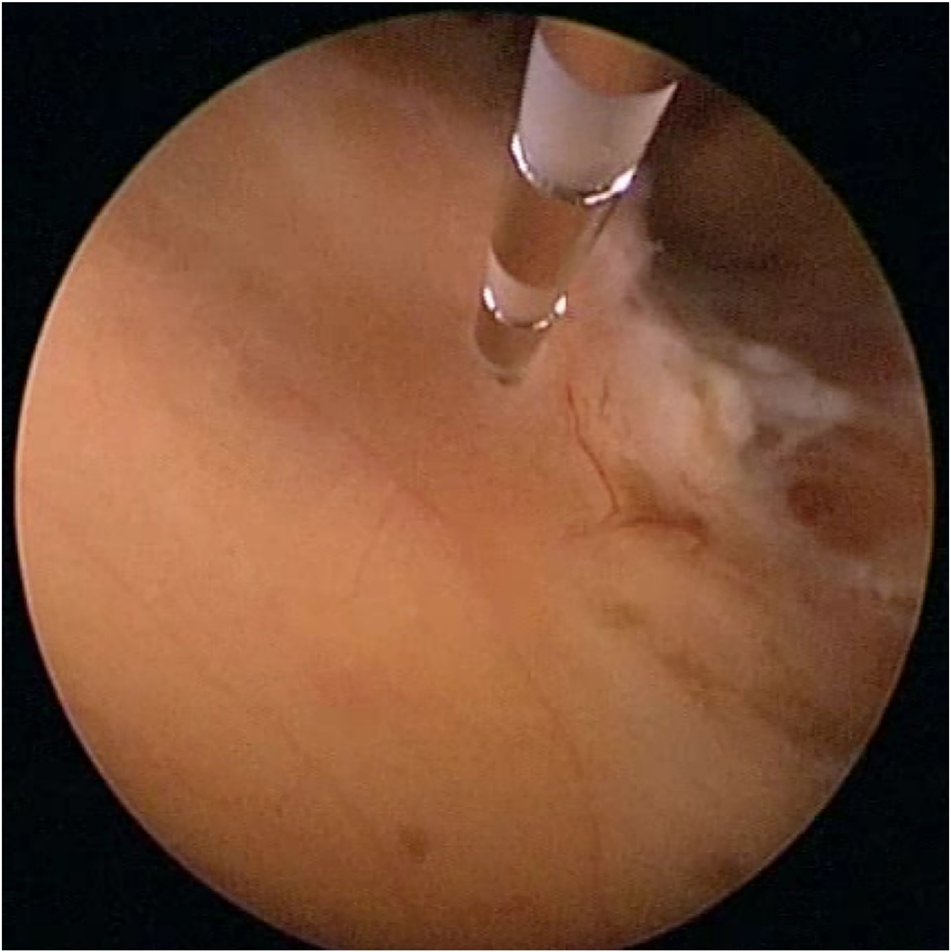
Intraventricular stereotactic electroencephalography (SEEG) electrode implantation under ventriculoscope.

SEEG was developed for the invasive mapping of refractory focal epilepsy. SEEG can identify seizure sites deep in the brain that could not be detected by conventional scalp EEG and accurately locate the origin of seizures. Different from traditional SEEG that needs to drill multiple holes on the skull to implant electrodes, intraventricular SEEG is performed through the instrument channel of the ventriculoscope, avoiding repeated skull drillings and transcortical punctures (Figure 1). We used eight‐channel electrodes, and only signals in the brain parenchyma were recorded to avoid the influence of the high conductivity of CSF on EEG recording. The process of electrode placement and EEG mapping is completed under the visual supervision of doctor to achieve more accuracy and precision. The contact laser does not affect intraoperative SEEG monitoring because it has no electrical stimulation. Contact laser ablation was applied after identifying the epileptogenic foci through intraventricular SEEG.

### 2.2 Patients

IS children with far from satisfactory response to antiepileptic medications were screened for ventriculoscopic laser ablation. They were mainly from the department of pediatrics and had undergone EEG examination before admission. The inclusion criteria contained poor response to first‐line treatments, obvious focal abnormalities near the ventricle on brain MRI, and patients under 2‐year old with developmental retardation. Patients with metabolic diseases and degenerative diseases were excluded. No child received surgical treatment before and ventriculoscopic surgery for children had been carried out skillfully in our hospital before admission. The study was approved by the institutional review board of the Chinese PLA General Hospital, and informed consent was obtained from each patient's parents.

### 2.3 Outcomes

In this study, seven IS children underwent intraventricular SEEG and laser ablation in our department, six were male and one was female. The median age was 9 months (range: 6 months to 1 year and 9 months). As to the form of epileptic seizures, six presented with nodding spasm, and one was absence seizures. Demographic information and disease characteristics of included patients are listed in Table 1. The structural changes were mainly encephalomalacia due to perinatal asphyxia, perinatal trauma, or spontaneous hemorrhage. The median number of kinds of anti‐IS drugs or antiepileptic drugs taken before surgery was 3.5 (range 3–6). Patients were followed up for a median time of 3.1 years (range 2.0–4.8 years). The Engel Epilepsy Surgery Outcome Scale was used to evaluate the surgical outcome (Chisolm et al., 2022). The Engel classification of each child is presented in Table 1: four (57%) in Engel class I, one (14%) in Engel class II, one (14%) in Engel class III, and one (14%) in Engel class IV at the last follow‐up. Overall, 86% (6/7) of the children got an Engel class ≤III epilepsy outcome. After surgery, five (71%) children had a reduction in seizure medication usage, and the other two were on the same amount as preablation. None of the children experienced serious new neurological deficit such as motor or speech deficits after surgery. No patient suffered peri or postoperative infection or died in the postoperative follow‐up. It is a pity that only two (29%) of seven children entered kindergarten normally. The developmental delay and intellectual disability were not fully addressed in all children.

**TABLE 1:** Characteristics of patients.

Pt No.	Sex	Age	Structural changes	Epileptic form	Medications	Engel Class at last follow‐up	Complications
1	F	7 months	Encephalomalacia in right frontal temporal parietal and insula lobes	Nodding spasm	ACTH, topiramate, clobazam, vitamin B6, clonazepam	I	None
2	M	9 months	Encephalomalacia after hemorrhage in left frontal temporal insula lobes	Nodding and hugging seizures	ACTH, topiramate, sodium valproate	II	None
3	M	7 month	Encephalomalacia in right temporal insular lobes	Nodding spasm	ACTH, topiramate, vitamin B6, phenobarbital	IV	None
4	M	9 months	Encephalomalacia in left temporal parietal lobes and basal ganglia area	Nodding spasm	Corticosteroids, topiramate, vitamin B12	III	None
5	M	9 months	Encephalomalacia after hemorrhage in right frontal parietal lobes	Nodding spasm, limb spasm	ACTH, sodium valproate, levetiracetam	I	None
6	M	12 months	Left paraventricular encephalomalacia, bilateral ventricles enlargement	Absence seizure, limb spasm	ACTH, topiramate, corticosteroids, sodium valproate, vitamin B6, B12	I	None
7	M	1 year 9 months	Left paraventricular encephalomalacia,	Nodding spasm	ACTH, topiramate, sodium valproate, levetiracetam	I	None

Abbreviations: ACTH, adrenocorticotropic hormone; F, female; M, male.

### 2.4 Illustrative cases

#### 2.4.1 Case 1

**FIGURE 2:**
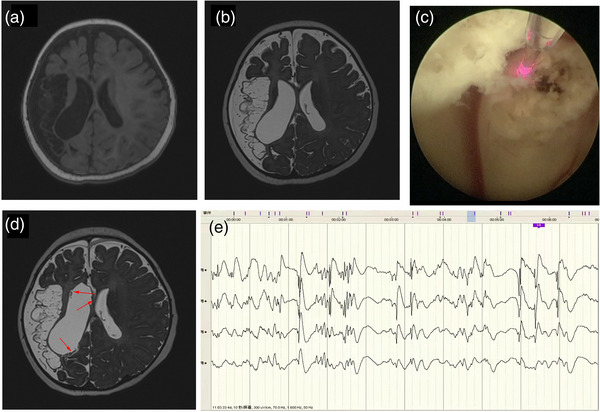
Illustrative case 1: (A) T1 image showing encephalomalacia foci in right frontal, temporal, parietal, and insula lobes; (B) T2 SPACE image showing encephalomalacia foci in right frontal, temporal, parietal, and insula lobes; (C) laser ablation under ventriculoscope; (D) T2 SPACE image after laser ablation; (E) intraoperative stereotactic electroencephalography (SEEG) demonstrated hypsarrhythmia. Red arrows refer to the area that has been ablated.

This 7‐month‐old baby girl was diagnosed with developmental delay 4 months ago due to inability to lift up. She suffered from nodding spasm 20 days before admission. The nervous system examination showed that she was unable to roll over, sit alone, grasp, or chase objects. No definite history of brain infarction or perinatal hypoxia was reported. Her brain MRI showed multiple encephalomalacia foci accompanied with gliosis in the right frontal, temporal, parietal, and insula lobes, localized brain atrophy, and the right thalamus, brain stem, and basal ganglia were smaller than those on the opposite side (Figure 2). The encephalomalacia foci were adjacent to the right lateral ventricle, creating possibilities for intraventricular SEEG and laser ablation. The preoperative EEG showed multifocal, generalized epileptiform discharges, hypsarrthmia. She was diagnosed with IS and treated with five different kinds of drugs, including ACTH, topiramate, clobazam, vitamin B6, and clonazepam. Nevertheless, the spasms still could not be well controlled. Given the above conditions, surgical treatment became an option.

In the surgery, the CURVE optical navigation system (Brainlab) was used to guide the process of ventricular puncture to improve the accuracy and reduce trauma. The ventriculoscope was inserted into the right lateral ventricle under the real‐time guidance of neuronavigation. Under the monitoring of ventriculoscope, an eight‐channel SEEG electrode was inserted into white matter under the ependyma of the frontal and occipital horn of right lateral ventricle to detect abnormal EEG. Abnormal epileptiform discharges were detected around the subependymal white matter of right frontal horn (Figure 2E). After SEEG tracing and confirmation, a contact laser fiber was inserted through the ventriculoscope working channel. The subependymal white matter of the frontal and occipital horn of right lateral ventricle with abnormal EEG was ablated using 15 Wt of power, and the ablation range was 3 × 3 cm^2^ with a depth about 5 mm (Figure 2D). We traced subependymal EEG through ventriculoscope working channel, avoiding multiple long‐distance punctures of the brain parenchyma. Apart from reducing trauma to brain, direct observation might contribute to the identification of epileptogenic foci with abnormal appearance and anatomy. We attempted to disrupt the epilepsy networks by laser ablation of detected abnormal regions. Postoperative EEG showed a significant reduction in abnormal discharges. The child took only topiramate with no more seizure attack after operation.

#### 2.4.2 Case 2

**FIGURE 3:**
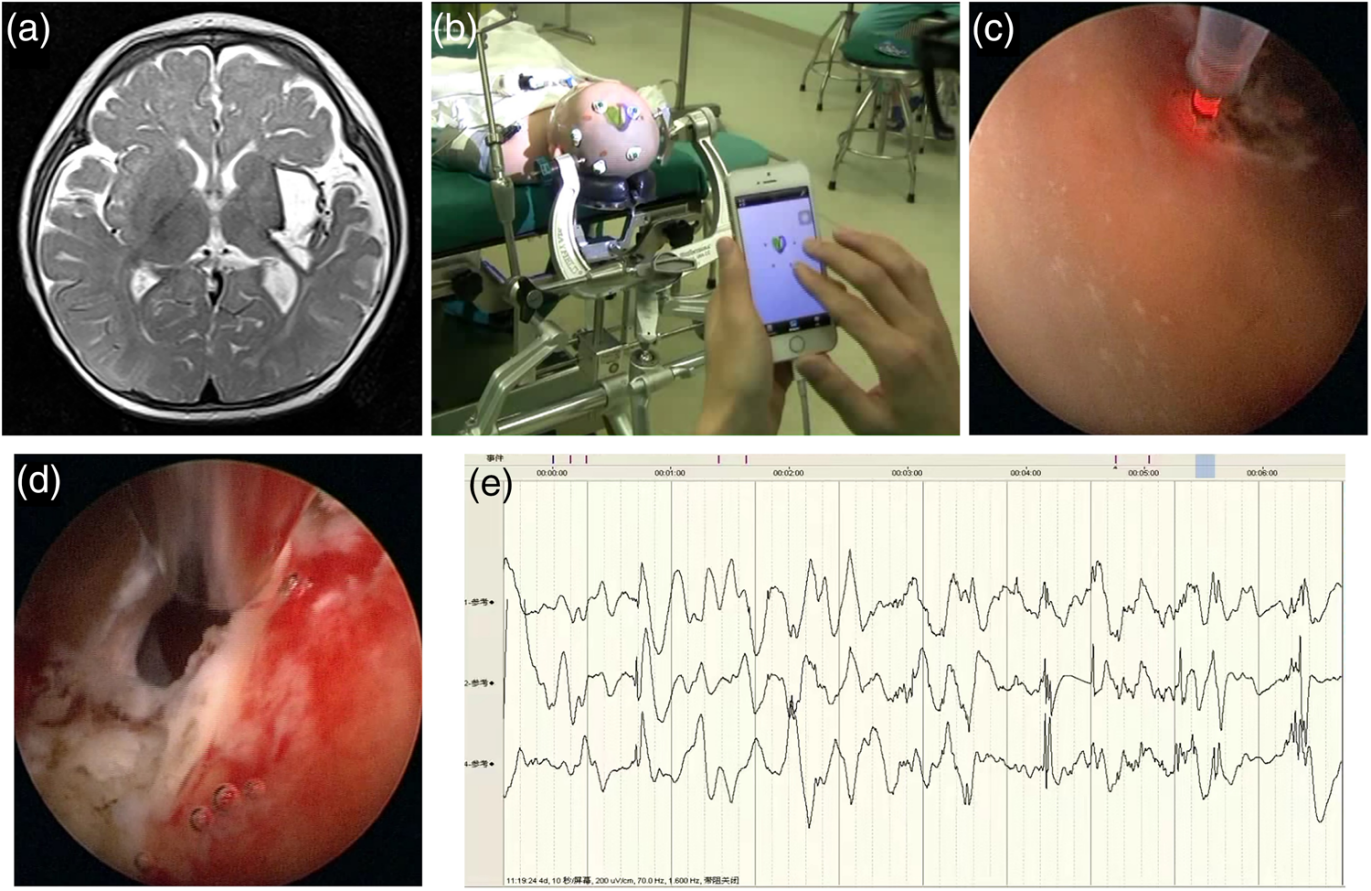
Illustrative case 2: (A) T2 image showing encephalomalacia foci after hemorrhage; (B) neuronavigation to locate the ventricle and guide the ventriculocentesis; (C) laser ablation under ventriculoscope; (D) ostomy between lateral ventricle and cystic cavity; (E) intraoperative stereotactic electroencephalography (SEEG) showed hypsarrhythmia.

A 9‐month‐old baby boy was admitted because of nodding and hugging seizures for 5 months. The cluster seizures occurred about four times per day, accompanied with crying. MRI demonstrated encephalomalacia after hemorrhage in left frontal temporal insula lobes. He was diagnosed with IS according to preoperative EEG manifested as hypsarrthmia. Seizure attack could not be prevented after the combined treatment of ACTH, topiramate, and sodium valproate. After careful evaluation, the patient underwent ventriculoscopic SEEG and laser ablation (Figure 3).

Neuronavigation was applied to guide the neuroendoscope to the encephalomalacia through the best puncture path. Hemosiderin deposition and a large number of cheese‐like substances referred a previous hemorrhage. Under the surveillance of ventriculoscope, an eight‐channel SEEG electrode was inserted into the left occipital lobe, temporal insula lobe, and the capsule wall near the hippocampus surrounding the encephalomalacia to detect abnormal epileptiform discharge. After locating the abnormal discharge site, we ablated the lateral wall of the encephalomalacia with the ablation range of 3 × 3 cm^2^ and the ablation depth of 5 mm using 15 Wt of power. In this case, we also made a 6 mm fistula with contact laser at the weakest part between the encephalomalacia and the left ventricle under navigation to avoid cyst expansion in the future (Figure 3D). The patient achieved seizure free at 3 days after operation.

## 3 DISCUSSION

The spectrum of IS includes epileptic seizures and cognitive and behavioral developmental disabilities (Pavone et al., 2020). Apart from well‐recognized structural, infectious, metabolic, and immunologic defects and genetic abnormalities, some etiologies of IS are still unknown (Scheffer et al., 2017). Different pathogenic events may concur in causing the IS. Many questions remain unclear and unsolved though considerable studies have been carried out and great advances in the field of genetics have been achieved. A better understanding of diagnosis, treatment, and prognosis of IS are warranted. Whatever, early recognition and intervention remain a priority for the optimal outcome of IS infants. Although several classical and novel antiepileptic drugs have been used as monotherapy or in combination for IS (Song et al., 2017), ACTH, corticosteroids, and vigabatrin, alone or in combination, remain the first‐line treatment.

Children refractory to drug therapy should be evaluated for surgery, especially if focal brain lesions are present (Buckley et al., 2016). Epileptogenic focus resection, multilobar resection or disconnection, hemispherotomy, corpus callosotomy, and vagus nerve stimulation are currently common surgical approaches. Early surgery is hypothesized to improve developmental outcome and quality of life in children with IS (Chipaux et al., 2017). The efficacy of surgery differs, highly depends on the etiology, especially whether there is a lesion on brain MRI (Barba et al., 2017; Chugani et al., 2015; Lee et al., 2014; Shan et al., 2021). Although the efficacy and safety of epilepsy surgery have been fully substantiated, the attendant risks of open surgery with regard to procedural morbidity and damage to adjacent brain tissue cannot be completely eliminated (Buckley et al., 2016). This calls for minimally invasive alternatives when treating certain epileptogenic lesions, especially those adjacent to or involving deep or eloquent brain structures.

Laser has been used in neurosurgery for more than 50 years. Since the lack of quality control and a real‐time monitoring system, the clinical use of laser was initially very limited. When the laser radiates to human tissue, complex light energy to heat energy effect thermocoagulative necrosis occurs, manifesting in the form of tissue gasification, condensation, and cutting. Compared with noncontact lasers, contact lasers are currently widely used in clinical practice. LITT was first described in brain tumor models by Bown (1983) in 1983. Traditional laser ablation or LITT stereotactically places the probe over the volume of tissue targeted for ablation through a hole that is drilled into the skull (Patel & Kim, 2020). There are two commonly used lasers, lasers with wavelengths of 1064 nm and lasers with wavelengths of 980 nm. After the first report to treat drug‐resistant epilepsy in 2012, LITT has been studied for treatment of mesial temporal lobe epilepsy, focal cortical dysplasia, hypothalamic hamartoma, cerebral cavernous malformation, heterotopia, and corpus callosotomy, achieving a satisfactory seizure‐free rate ranging from 53% to 93% (Curry et al., 2012; Kang et al., 2016; Satzer et al., 2020; Shimamoto et al., 2019; Shukla et al., 2017; Wicks et al., 2016). In pediatric epilepsy, LITT was reported to enable 16 out of 20 (80%) children with intractable insular epilepsy and 4 out of 5 (80%) children with periventricular nodular heterotopia‐related epilepsy had an Engel class ≤III at the last follow‐up (Perry et al., 2017; Ravindra et al., 2021). We attempted to relieve seizure in IS children through ventriculoscopic SEEG and ventriculoscopic laser ablation, a way in which repeated skull drillings and transcortical punctures were avoided. In our study, six out of seven (86%) children with IS achieved an Engel class ≤III epilepsy at the final follow‐up. The efficacy was similar to that of LITT in treating children with intractable epilepsy. Compared with traditional open surgery, laser ablation showed advantages in the protection of nearby critical structures and minimal patient discomfort.

Different from LITT, the contact laser we used reached the target through the instrument channel of the ventriculoscope rather than through stereotactic puncture. We ablated the suspected epileptogenic foci detected by SEEG in the underwater environment, avoiding excessive damage to normal brain tissue by high temperatures. The whole process of ablation was completed under the monitoring of ventriculoscope to better observe the ablation effect and avoid excessive or insufficient ablation. To our knowledge, this was the first study reported to perform iventriculoscopic SEEG and ventriculoscopic laser ablation to treat IS patients, which not only ensured the accuracy of electrode placement but also avoided the risk of injury and bleeding caused by repeated brain parenchyma puncture. The laser wavelength used was 1064 nm, the energy of which followed the Gaussian distribution with the center energy concentrated and the peripheral gradient energy descended.

The specific mechanism by which laser ablation results in seizure freedom remains unclear. Based on our experience, laser ablation might result in seizure freedom by destroying the local brain network and blocking the spread of abnormal discharges. The energy from the laser light was converted to heat within the target volume, inducing a cascade of enzymes that lead to protein denaturation, membrane dissolution, and vessel sclerosis, all precursors of necrosis. As a minimally invasive technique, ventriculoscopic laser ablation shows promising efficacy in alleviating epileptic seizures in children with IS. All patients included in this study had at least one structural change on MRI. This was the anatomical basis on which ventriculoscopic laser ablation could be effective. Periventricular heterotopia, or subependymal heterotopia, which means a cluster of ectopic gray matter along the ventricle because of abnormal neuronal migration during brain development, was not discovered in the case series we studied. The structural changes in our series were mainly encephalomalacia due to perinatal asphyxia, perinatal trauma, or spontaneous hemorrhage.

This is a retrospective technical report with limited sample size to verify the feasibility and efficacy of intraventricular SEEG and laser ablation for the treatment of IS. The findings in our study support the feasibility, efficacy, and safety of intraventricular laser ablation for the treatment of IS, with seizure outcomes similar to those of open surgery. Intraventricular laser ablation provides a minimally invasive treatment option for children with IS.

## CONFLICT OF INTREST STATEMENT

All authors have no conflicts of interest to disclose.

### PEER REVIEW

The peer review history for this article is available at https://publons.com/publon/10.1002/brb3.3184.

## Data Availability

The data that support the findings of this study are available from the corresponding author upon reasonable request.

## References

[brb33184-bib-0001] Barba, C. , Mai, R. , Grisotto, L. , Gozzo, F. , Pellacani, S. , Tassi, L. , Francione, S. , Giordano, F. , Cardinale, F. , & Guerrini, R. (2017). Unilobar surgery for symptomatic epileptic spasms. Annals of Clinical and Translational Neurology, 4, 36–45. 10.1002/acn3.373 28078313 PMC5221449

[brb33184-bib-0002] Bown, S. G. (1983). Phototherapy in tumors. World Journal of Surgery, 7(6), 700–709. 10.1007/BF01655209 6419477

[brb33184-bib-0003] Buckley, R. , Estronza‐Ojeda, S. , & Ojemann, J. G. (2016). Laser ablation in pediatric epilepsy. Neurosurgery Clinics of North America, 27, 69–78. 10.1016/j.nec.2015.08.006 26615109

[brb33184-bib-0004] Chipaux, M. , Dorfmüller, G. , Fohlen, M. , Dorison, N. , Metten, M.‐A. , Delalande, O. , Ferrand‐Sorbets, S. , & Taussig, D. (2017). Refractory spasms of focal onset—A potentially curable disease that should lead to rapid surgical evaluation. Seizure: The Journal of the British Epilepsy Association, 51, 163–170. 10.1016/j.seizure.2017.08.010 28873364

[brb33184-bib-0005] Chisolm, P. F. , Warner, J. D. , Hale, A. T. , Estevez‐Ordonez, D. , Murdaugh, D. , Rozzelle, C. J. , & Blount, J. P. (2022). Quantifying and reporting outcome measures in pediatric epilepsy surgery: A systematic review. Epilepsia, 63, 2754–2781. 10.1111/epi.17369 35847999

[brb33184-bib-0006] Chopra, S. S. (2020). Infantile spasms and West syndrome—A clinician's perspective. Indian Journal of Pediatrics, 87, 1040–1046. 10.1007/s12098-020-03279-y 32557136

[brb33184-bib-0007] Chugani, H. T. , Ilyas, M. , Kumar, A. , Juhász, C. , Kupsky, W. J. , Sood, S. , & Asano, E. (2015). Surgical treatment for refractory epileptic spasms: The Detroit series. Epilepsia, 56, 1941–1949. 10.1111/epi.13221 26522016 PMC4679547

[brb33184-bib-0008] Curry, D. J. , Gowda, A. , Mcnichols, R. J. , & Wilfong, A. A. (2012). MR‐guided stereotactic laser ablation of epileptogenic foci in children. Epilepsy & Behavior, 24, 408–414. 10.1016/j.yebeh.2012.04.135 22687387

[brb33184-bib-0009] Kang, J. Y. , Wu, C. , Tracy, J. , Lorenzo, M. , Evans, J. , Nei, M. , Skidmore, C. , Mintzer, S. , Sharan, A. D. , & Sperling, M. R. (2016). Laser interstitial thermal therapy for medically intractable mesial temporal lobe epilepsy. Epilepsia, 57, 325–334. 10.1111/epi.13284 26697969

[brb33184-bib-0010] Kelley, S. A. , & Knupp, K. G. (2018). Infantile spasms—Have we made progress? Current Neurology and Neuroscience Reports, 18, 27. 10.1007/s11910-018-0832-8 29671077

[brb33184-bib-0011] Lee, Y.‐J. , Kim, E.‐H. , Yum, M.‐S. , Lee, J. K. , Hong, S. , & Ko, T.‐S. (2014). Long‐term outcomes of hemispheric disconnection in pediatric patients with intractable epilepsy. Journal of Clinical Neurology, 10, 101–107. 10.3988/jcn.2014.10.2.101 24829595 PMC4017012

[brb33184-bib-0012] Lewis, E. C. , Weil, A. G. , Duchowny, M. , Bhatia, S. , Ragheb, J. , & Miller, I. (2015). MR‐guided laser interstitial thermal therapy for pediatric drug‐resistant lesional epilepsy. Epilepsia, 56, 1590–1598. 10.1111/epi.13106 26249524

[brb33184-bib-0013] Lux, A. L. , Edwards, S. W. , Hancock, E. , Johnson, A. L. , Kennedy, C. R. , Newton, R. W. , O'callaghan, F. J. K. , Verity, C. M. , & Osborne, J. P. (2004). The United Kingdom Infantile Spasms Study comparing vigabatrin with prednisolone or tetracosactide at 14 days: A multicentre, randomised controlled trial. Lancet, 364, 1773–1778. 10.1016/S0140-6736(04)17400-X 15541450

[brb33184-bib-0014] O'callaghan, F. J. K. , Edwards, S. W. , Alber, F. D. , Hancock, E. , Johnson, A. L. , Kennedy, C. R. , Likeman, M. , Lux, A. L. , Mackay, M. , Mallick, A. A. , Newton, R. W. , Nolan, M. , Pressler, R. , Rating, D. , Schmitt, B. , Verity, C. M. , & Osborne, J. P. (2017). Safety and effectiveness of hormonal treatment versus hormonal treatment with vigabatrin for infantile spasms (ICISS): A randomised, multicentre, open‐label trial. Lancet Neurology, 16, 33–42. 10.1016/S1474-4422(16)30294-0 27838190

[brb33184-bib-0015] Osborne, J. P. , Lux, A. L. , Edwards, S. W. , Hancock, E. , Johnson, A. L. , Kennedy, C. R. , Newton, R. W. , Verity, C. M. , & O'callaghan, F. J. K. (2010). The underlying etiology of infantile spasms (West syndrome): Information from the United Kingdom Infantile Spasms Study (UKISS) on contemporary causes and their classification. Epilepsia, 51, 2168–2174. 10.1111/j.1528-1167.2010.02695.x 20726878

[brb33184-bib-0016] Patel, B. , & Kim, A. H. (2020). Laser interstitial thermal therapy. Missouri Medicine, 117, 50–55. 32158050 PMC7023945

[brb33184-bib-0017] Pavone, P. , Polizzi, A. , Marino, S. D. , Corsello, G. , Falsaperla, R. , Marino, S. , & Ruggieri, M. (2020). West syndrome: A comprehensive review. Neurological Sciences, 41, 3547–3562. 10.1007/s10072-020-04600-5 32827285 PMC7655587

[brb33184-bib-0018] Pavone, P. , Striano, P. , Falsaperla, R. , Pavone, L. , & Ruggieri, M. (2014). Infantile spasms syndrome, West syndrome and related phenotypes: What we know in 2013. Brain & Development, 36, 739–751. 10.1016/j.braindev.2013.10.008 24268986

[brb33184-bib-0019] Perry, M. S. , Donahue, D. J. , Malik, S. I. , Keator, C. G. , Hernandez, A. , Reddy, R. K. , Perkins, F. F. , Lee, M. R. , & Clarke, D. F. (2017). Magnetic resonance imaging‐guided laser interstitial thermal therapy as treatment for intractable insular epilepsy in children. Journal of Neurosurgery: Pediatrics, 20, 575–582. 10.3171/2017.6.PEDS17158 29027866

[brb33184-bib-0020] Ravindra, V. M. , Lee, S. , Gonda, D. , Patino, I. , Ruggieri, L. , Ikeda, D. S. , & Curry, D. J. (2021). Magnetic resonance‐guided laser interstitial thermal therapy for pediatric periventricular nodular heterotopia‐related epilepsy. Journal of Neurosurgery: Pediatrics, 28, 657–662. 10.3171/2021.5.PEDS21171 34560627

[brb33184-bib-0021] Satzer, D. , Tao, J. X. , Issa, N. P. , Chen, Z. , Wu, S. , Rose, S. , Collins, J. , Awad, I. A. , & Warnke, P. C. (2020). Stereotactic laser interstitial thermal therapy for epilepsy associated with solitary and multiple cerebral cavernous malformations. Neurosurgical Focus [Electronic Resource], 48, E12. 10.3171/2020.1.FOCUS19866 32234994

[brb33184-bib-0022] Scheffer, I. E. , Berkovic, S. , Capovilla, G. , Connolly, M. B. , French, J. , Guilhoto, L. , Hirsch, E. , Jain, S. , Mathern, G. W. , Moshé, S. L. , Nordli, D. R. , Perucca, E. , Tomson, T. , Wiebe, S. , Zhang, Y.‐H. , & Zuberi, S. M. (2017). ILAE classification of the epilepsies: Position paper of the ILAE Commission for Classification and Terminology. Epilepsia, 58, 512–521. 10.1111/epi.13709 28276062 PMC5386840

[brb33184-bib-0023] Shan, W. , Mao, X. , Wang, X. , Hogan, R. E. , & Wang, Q. (2021). Potential surgical therapies for drug‐resistant focal epilepsy. CNS Neuroscience & Therapeutics, 27, 994–1011. 10.1111/cns.13690 34101365 PMC8339538

[brb33184-bib-0024] Shimamoto, S. , Wu, C. , & Sperling, M. R. (2019). Laser interstitial thermal therapy in drug‐resistant epilepsy. Current Opinion in Neurology, 32, 237–245. 10.1097/WCO.0000000000000662 30694919

[brb33184-bib-0025] Shukla, N. , Ho, A. L. , Pendharkar, A. , Sussman, E. , & Halpern, C. (2017). Laser interstitial thermal therapy for the treatment of epilepsy: Evidence to date. Neuropsychiatric Disease and Treatment, 13, 2469–2475. 10.2147/NDT.S139544 29026310 PMC5627747

[brb33184-bib-0026] Song, J. M. , Hahn, J. , Kim, S. H. , & Chang, M. J. (2017). Efficacy of treatments for infantile spasms: A systematic review. Clinical Neuropharmacology, 40, 63–84. 10.1097/WNF.0000000000000200 28288483

[brb33184-bib-0027] Specchio, N. , Pietrafusa, N. , Ferretti, A. , De Palma, L. , Santarone, M. E. , Pepi, C. , Trivisano, M. , Vigevano, F. , & Curatolo, P. (2020). Treatment of infantile spasms: Why do we know so little? Expert Review of Neurotherapeutics, 20, 551–566. 10.1080/14737175.2020.1759423 32316776

[brb33184-bib-0028] West, W. J. (1841). On a peculiar form of infantile convulsions. Lancet, 35, 724–725. 10.1016/S0140-6736(00)40184-4

[brb33184-bib-0029] Wicks, R. T. , Jermakowicz, W. J. , Jagid, J. R. , Couture, D. E. , Willie, J. T. , Laxton, A. W. , & Gross, R. E. (2016). Laser interstitial thermal therapy for mesial temporal lobe epilepsy. Neurosurgery, 79, S83–S91. 10.1227/NEU.0000000000001439 27861328

[brb33184-bib-0030] Wilmshurst, J. M. , Ibekwe, R. C. , & O'callaghan, F. J. K. (2017). Epileptic spasms‐175 years on: Trying to teach an old dog new tricks. Seizure: The Journal of the British Epilepsy Association, 44, 81–86. 10.1016/j.seizure.2016.11.021 27989601

